# MDM2 is a potential therapeutic target and prognostic factor for ovarian clear cell carcinomas with wild type TP53

**DOI:** 10.18632/oncotarget.12175

**Published:** 2016-09-21

**Authors:** Chinami Makii, Katsutoshi Oda, Yuji Ikeda, Kenbun Sone, Kosei Hasegawa, Yuriko Uehara, Akira Nishijima, Kayo Asada, Takahiro Koso, Tomohiko Fukuda, Kanako Inaba, Shinya Oki, Hidenori Machino, Machiko Kojima, Tomoko Kashiyama, Mayuyo Mori-Uchino, Takahide Arimoto, Osamu Wada-Hiraike, Kei Kawana, Tetsu Yano, Keiichi Fujiwara, Hiroyuki Aburatani, Yutaka Osuga, Tomoyuki Fujii

**Affiliations:** ^1^ Department of Obstetrics and Gynecology, Graduate School of Medicine, The University of Tokyo, Tokyo, Japan; ^2^ Department of Gynecologic Oncology, Saitama Medical University International Medical Center, Saitama, Japan; ^3^ Division of Genome Science, Research Center for Advanced Science and Technology, The University of Tokyo, Tokyo, Japan; ^4^ Department of Obstetrics and Gynecology, National Center for Global Health and Medicine, Tokyo, Japan

**Keywords:** MDM2, TP53, prognosis, molecular-targeted therapy, ovarian clear cell carcinoma

## Abstract

MDM2, a ubiquitin ligase, suppresses wild type TP53 via proteasome-mediated degradation. We evaluated the prognostic and therapeutic value of MDM2 in ovarian clear cell carcinoma. *MDM2* expression in ovarian cancer tissues was analyzed by microarray and real-time PCR, and its relationship with prognosis was evaluated by Kaplan-Meier method and log-rank test. The anti-tumor activities of *MDM2* siRNA and the MDM2 inhibitor RG7112 were assessed by cell viability assay, western blotting, and flow cytometry. The anti-tumor effects of RG7112 *in vivo* were examined in a mouse xenograft model. *MDM2* expression was significantly higher in clear cell carcinoma than in ovarian high-grade serous carcinoma (*P* = 0.0092) and normal tissues (*P* = 0.035). High *MDM2* expression determined by microarray was significantly associated with poor progression-free survival and poor overall survival (*P* = 0.0002, and *P* = 0.0008, respectively). Notably, RG7112 significantly suppressed cell viability in clear cell carcinoma cell lines with wild type *TP53*. RG7112 also strongly induced apoptosis, increased TP53 phosphorylation, and stimulated expression of the proapoptotic protein PUMA. Similarly, siRNA knockdown of *MDM2* induced apoptosis. Finally, RG7112 significantly reduced the tumor volume of xenografted RMG-I clear cell carcinoma cells (*P* = 0.033), and the density of microvessels (*P* = 0.011). Our results highlight the prognostic value of *MDM2* expression in clear cell carcinoma. Thus, MDM2 inhibitors such as RG7112 may constitute a class of potential therapeutics.

## INTRODUCTION

Ovarian clear cell carcinoma is one of four major histological types of epithelial ovarian cancer, the others being serous, mucinous, and endometrioid carcinomas [1–3]. Of these, high-grade serous carcinoma is the most common, and accounts for > 50-60% of cases [4, 5]. Thus, most genome-wide analyses, including whole-exome sequencing, target this form of ovarian cancer [6–8]. However, the ratio of clear cell carcinoma is high in Asia, especially in Japan, where it constitutes approximately 25% of cases [2]. Notably, clear cell carcinomas are characteristically resistant to platinum-based chemotherapy [9], and the 3-year survival rate in patients with ≥ 2 cm residual tumor is only 10.2%, which is significantly poorer than the 45.9% survival rate in patients with high-grade serous cancer [9]. Only a few molecular targeted therapies have been approved for ovarian cancers, none of which is specific for clear cell carcinoma. Therefore, novel therapeutic strategies are needed against this form of ovarian cancer.

*TP53* mutations are characteristically infrequent, and are present in only 10% of ovarian clear cell carcinomas, with loss of heterozygosity in < 20% [10–12]. In contrast, *TP53* mutations are present in 96% of high-grade serous tumors [6]. TP53 is a key tumor suppressor that induces cell cycle arrest, apoptosis, autophagy, and senescence while inhibiting angiogenesis and metastasis [13–15]. Notably, TP53 activity is determined not only by abundance, but also by phosphorylation. For instance, TP53 is activated by phosphorylation at Ser-46 to induce expression of apoptosis genes such as *PUMA* and *TP53AIP1* in response to severe DNA damage or extreme TP53 overexpression [16]. TP53 activation also inhibits angiogenesis via suppression of hypoxia-inducible factor 1alpha (HIF-1a) [17]. Therefore, TP53 is expected to function as a tumor suppressor in cancers with wild type *TP53*, including clear cell carcinoma. Nevertheless, TP53 may be inactivated even in the absence of mutations, and this inactivation may promote cell survival in some of these cancers [18, 19].

Cellular TP53 levels are regulated by degradation pathways that require E3 ubiquitin ligases [20, 21]. Of these, murine double minute 2 (MDM2) and MDM4 (also known as MDMX, a structural homolog of MDM2) are the most essential [22]. Indeed, TP53 inactivation by overexpression of MDM2 and other E3 ligases has been reported in human cancers [23]. In addition, *TP53* mutations are inversely correlated with abundant *MDM2* expression [24]. In this light, MDM2 inhibitors such as Nutlin-3a and RG7112 were developed recently to block the interaction between TP53 and MDM2, and thereby stabilize TP53. Importantly, these compounds were reported to have *in vitro* and *in vivo* antitumor activity in human cancers with wild type TP53 [25–28], and are now in early-phase clinical trials [29–31]. Nevertheless, whether MDM2 and/or MDM4 are overexpressed in clear cell carcinoma remains to be established, along with whether MDM2 inhibitors are active against these forms of cancer. In this study, we investigated the expression of MDM2 and MDM4 in clear cell carcinomas, and evaluated the *in vitro* and *in vivo* activity of the MDM2 inhibitor RG7112 against clear cell tumors with wild type TP53.

## RESULTS

### High *MDM2* expression is significantly associated with clear cell carcinoma histology and poor prognosis

**Figure 1:**
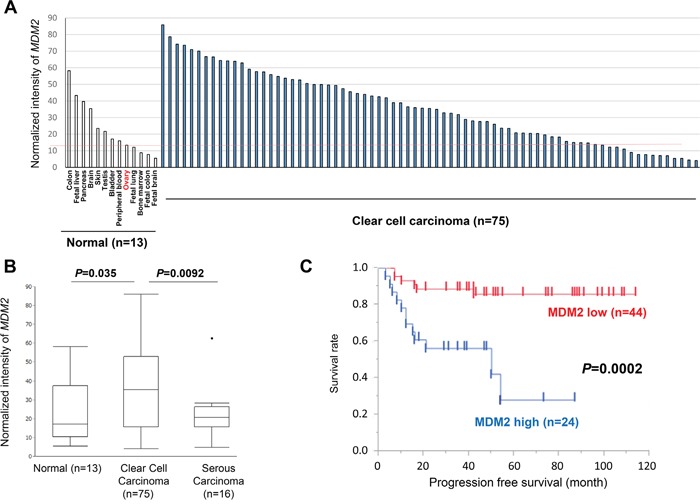
Expression of MDM2 in normal tissues and ovarian cancers **A.** Expression of *MDM2* in 13 normal tissues and 75 ovarian clear cell carcinomas, as determined by microarray analysis. **B.** Comparison (t-test) of *MDM2* expression in normal tissues, clear cell, and high-grade serous carcinomas. **C.** Survival analysis (progression-free survival) using Kaplan-Meier method and log-rank test in clear cell carcinomas without *TP53* mutations (n = 68). The upper 1/3 among clear cell carcinomas was defined as “MDM2 high” on the basis of the expression level determined by microarray.

*MDM2* mRNA expression was analyzed by microarray in 75 clear cell carcinomas, 13 normal tissues, and 16 high-grade serous ovarian cancers. MDM2 expression was higher in 61 of 75 (81%) clear cell carcinomas than in normal ovarian tissue (Figure 1A and Supplementary Table 1). Indeed, *MDM2* expression was significantly higher in clear cell carcinomas than in normal tissues (*P* = 0.035) and high-grade serous carcinomas (*P* = 0.0092, Figure 1B). However, expression of *MDM4* was significantly lower in both cancer tissues than in normal tissues (Supplementary Figure 1A). Clear cell carcinomas were further stratified as MDM2-high (n = 25), MDM2-intermediate (n = 25), and MDM2-low (n = 25). *TP53* mutations were detected by Sanger sequencing in 4 (5.6%) clear cell carcinomas (Supplementary Figure 1B), all of which were MDM2-low or intermediate (Supplementary Table 1). In clear cell carcinomas without *TP53* mutations, high *MDM2* expression was significantly associated with poor progression-free survival (PFS) (*P* = 0.0002 by log-rank test, Figure 1C), as was advanced stage (*P* = 0.0002 by log-rank test, Supplementary Figure 1C), but not age (Supplementary Figure 1D). *MDM2*-high was also associated with poor overall survival (OS) (*P* = 0.0008) (Supplementary Figure 2A). The prognosis (either PFS or OS) was comparable between MDM2-intermediate and MDMs-low (Supplementary Figure 2B and 2C). Similarly, univariate analysis demonstrated that advanced stage (HR = 5.05, 95% CI = 1.84-12.91, *P* = 0.0025) and high *MDM2* expression (HR = 5.48, 95% CI = 2.10-15.97, *P* = 0.0005) were significantly associated with poor PFS (Table 1: upper rows) and with poor OS (Table 1: lower rows). In addition, multivariate analysis indicated that high *MDM2* expression was a poor prognostic factor for PFS (HR = 5.61, 95% CI = 2.11-16.62, *P* = 0.0005) and OS (HR = 6.14, 95% CI = 1.85-24.32, *P* = 0.0028, independent of age and cancer stage (Table 1). We also performed real-time PCR in 4 normal ovarian tissues and 17 of the 75 clear cell carcinomas (Supplementary Figure 3A), and found that *MDM2* expression was significantly higher in clear cell carcinomas than in normal ovaries (*P* = 0.039) (Supplementary Figure 3A), and that the expression level of *MDM2* determined by microarray was highly associated with that determined by real-time PCR (Supplementary Figure 3B).

**Table 1:** Univariate/multivariate analysis in CCC by *MDM2* expression, age and stage

Characteristics		Univariate			Multivariate		
		HR	95%CI	P-value	HR	95%CI	P-value
Age	≥50	1.45	0.52-5.13	0.5	0.97	0.32-3.55	0.95
	<50 (ref)						
Stage	III/IV	5.05	1.84-12.91	0.0025	5.16	1.8-14.2	0.0032
	I/II (ref)						
MDM2	High	5.48	2.1-15.97	0.0005	5.61	2.11-16.62	0.0005
	Low (ref)						
**Overall Survival**							

Characteristics		Univariate			Multivariate		
		HR	95%CI	P-value	HR	95%CI	P-value
Age	≥50	1.26	0.38-5.61	0.72	0.52	0.12-2.6	0.4
	<50 (ref)						
Stage	III/IV	8.6	2.83-26.99	0.0003	9.81	2.81-38.94	0.0004
	I/II (ref)						
MDM2	High	6.078	1.95-22.76	0.0018	6.14	1.85-24.32	0.0028
	Low (ref)						

**Figure 2:**
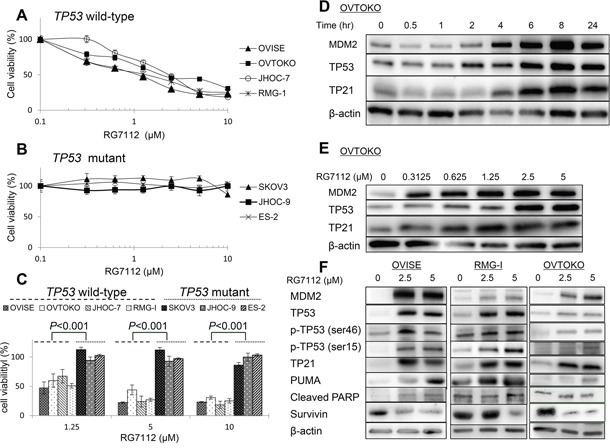
Cell viability and activation of TP53 target proteins in clear cell carcinomas treated with RG7112 **A** and **B.** Cell viability, as measured by MTT assay, in cells with wild type (A) or mutant (B) TP53. Experiments were repeated three times. **C.** Differences in cell viability in cell lines with wild type or mutant TP53 and exposed to indicated concentrations of RG7112. Group means were compared by paired t-test. **D** and **E.** Time- (D) and dose-dependent (E) accumulation of MDM2, TP53, and TP21, as measured by western blotting. RG7112 was added at 2.5 μM in (D). **F.** Induction of TP53 phosphorylation and TP53 target proteins (TP21 and PUMA), as determined by western blotting. Cleaved PARP and survivin were also assessed to detect proapoptotic signaling.

### RG7112 inhibits cell viability in clear cell carcinomas with wild type *TP53*

MDM2 expression was assessed by immunoblotting in 7 clear cell carcinoma cell lines with wild-type (OVISE, OVTOKO, JHOC-7, and RMG-I) or mutated (SKOV3, JHOC-9, and ES-2) *TP53* (Supplementary Figure 4). MDM2 expression was significantly higher in 3 of 4 *TP53* mutant cell lines than in the control (immortalized cells derived from ovarian surface epithelium).

Next, the antiproliferative effect of RG7112, an MDM2 inhibitor, was evaluated in these clear cell carcinomas. RG7112 suppressed cell proliferation in dose-dependent fashion only in cells with wild type *TP53*, with half maximal inhibitory concentration (IC_50_) between 1.0 and 2.2 μM (Figure 2A). On the other hand, IC_50_ was > 10 μM in cell lines with mutant *TP53* (Figure 2B). Indeed, differences in cell viability were statistically significant between tumors with wild type and mutated TP53 (*P* < 0.001 by paired t-test, Figure 2C). We also confirmed that the IC_50_ was > 10 μM in normal cell lines (COS-7 and 293T) (Supplementary Figure 5).

RG7112 increased the abundance of MDM2 and TP53 in OVTOKO cells in a time-dependent (at 2.5 μM) and dose-dependent fashion (Figure 2D and 2E), suggesting that interference with MDM2-TP53 binding resulted in accumulation of both proteins. Accordingly, expression of TP21, a well-known TP53 target gene, increased in time- and dose-dependent manner (Figure 2D and 2E). In particular, expression of TP53 and TP21 was induced within 2-4 h at RG7112 doses > 0.625 μM (Figure 2D and 2E). RG7112 also stimulated TP53-dependent apoptotic signaling in OVISE, RMG-I, and OVTOKO cells, as indicated by increased TP53 phosphorylation at Ser-46 and Ser-15, increased expression of the proapoptotic gene PUMA, and suppression of the antiapoptotic protein survivin (Figure 2F). Consequently, cleaved PARP accumulated following exposure to 2.5 or 5 μM RG7112 in OVISE cells (Figure 2F).

### RG7112 and *MDM2* siRNA induce apoptotic cell death in clear cell carcinoma with wild type TP53

**Figure 3:**
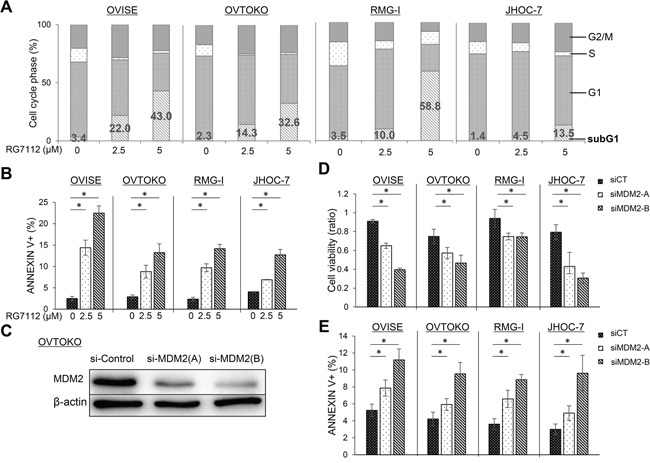
Induction of apoptosis after RG7112 treatment and MDM2 knockdown in clear cell carcinomas with wild type TP53 **A.** Cell cycle distribution after RG7112 treatment, as assessed by fluorescence-activated cell sorting. **B.** Apoptosis after RG7112 treatment, as evaluated by annexin V staining. **C.** siRNA knockdown of MDM2 in OVTOKO cells using MDM2-A and MDM2-B, as confirmed by western blotting. **D.** Cell viability after *MDM2* knockdown was assessed by MTT assay. **E.** Apoptosis after *MDM2* knockdown was assessed by annexin V assay. All experiments were repeated three times.

To further examine whether activation of TP53 is associated with RG7112-induced cell death, we analyzed the cell cycle distribution of clear cell carcinomas with wild type TP53 and exposed to RG7112 for 72 h. The compound expanded the sub-G1 population in dose-dependent manner (Figure 3A). In particular, the proportion of cells in sub-G1 increased to 14-59% in cells treated with 5 μM RG7112, while the S phase population contracted. In addition, exposure to 2.5 μM or 5 μM RG7112 significantly increased the ratio of apoptotic cells by 12-22%, as measured by annexin V staining (Figure 3B). Suppression of MDM2 expression by siRNAs (Figure 3C) also significantly suppressed cell viability (Figure 3D) and increased apoptotic cell death, as measured by MTT assay and annexin V staining, respectively (Figure 3E).

### *In vivo* antitumor activity of RG7112

**Figure 4:**
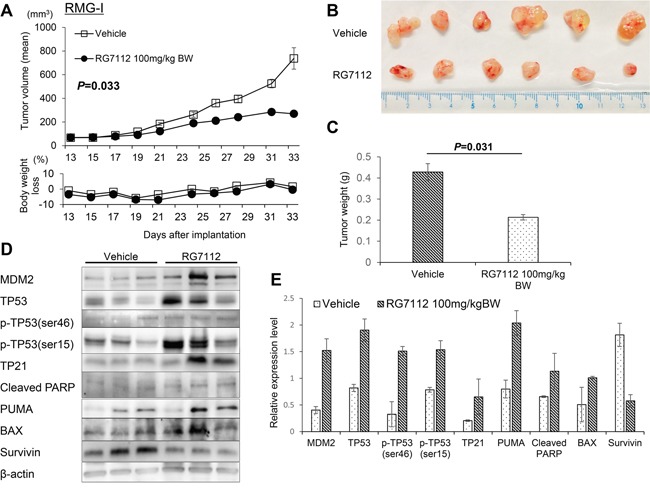
*In vivo* evaluation of RG7112 efficacy using xenografted RMG-I cells **A-C.** Tumor size and body weight in xenograft mouse models orally treated with RG7112 were measured after the start of RG7112 treatment 13 days post implantation (A). Tumors were collected from mice sacrificed after 21 days of RG7112 treatment (B). Tumor weights were compared by t-test between control and RG7112-treated animals (C). **D.** TP53 phosphorylation and expression of TP53 target proteins were compared between tumors from control and RG7112-treated animals (n = 3 per group). **E.** Expression of each protein relative to beta-actin was quantified in Image J. All experiments were repeated thrice.

To examine the antitumor activity of RG7112 *in vivo*, we established subcutaneous tumor xenograft models in nude mice by using RMG-I and OVISE cells. Each mouse was treated daily for three weeks with an oral dose of 100 mg/kg RG7112 or vehicle. RG7112 significantly suppressed the growth of xenografted RMG-I cells (*P* = 0.033 by t-test, Figure 4A-4C) and tended to suppress the growth of the OVISE cells, although the growth suppression in OVISE cells did not reach statistical significance (*P* = 0.061, Supplementary Figure 6A). We note that mice lost < 10% body weight throughout the study (Figure 4A and Supplementary Figure 6A). Western blotting of three tumors from RG7112-treated mice showed upregulation of MDM2, TP53, and TP53 phosphorylation at Ser15 and Ser46 (Figure 4D and 4E, and Supplementary Figure 6B and 6C). In addition, RG7112 also induced TP53 target genes such as TP21, PUMA, and BAX. In agreement with *in vitro* results, cleaved PARP accumulated while survivin diminished with RG7112 treatment (Figure 4D and 4E, and Supplementary Figure 6B and 6C).

### RG7112 suppresses HIF-1alpha in hypoxic conditions and reduces tumor vascularity

**Figure 5:**
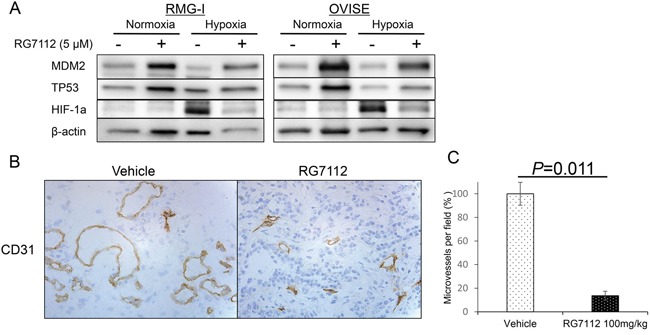
RG7112-induced suppression of HIF-1alpha during hypoxia and reduction of tumor vascularity **A.** Normoxic and hypoxic (under 1% O_2_) expression of MDM2, p53, and HIF-1alpha in RMG-I and OVISE cells treated with or without 5 μM RG7112. **B.** Immunohistochemical staining of CD31 to detect microvessels in RMG-I tumors from control and RG7112-treated mice. **C.** Microvessels per field was calculated in RMG-I tumors from control and RG7112-treated mice, and compared by t-test.

Finally, we examined whether RG7112 affects HIF-1alpha expression and tumor vascularity in clear cell carcinomas. Exposure to RG7112 (Figure 5A) and MDM2 siRNA (Supplementary Figure 7A) suppressed hypoxia (1% O_2_)-induced expression of HIF-1alpha in two cell lines with wild type TP53. In addition, large vessels stained with the endothelial marker CD31 were observed in tumor sections from control mice, whereas only small vessels were observed in RG7112-treated mice (Figure 5B and Supplementary Figure 7B). Indeed, the number of microvessels in RMG-I and OVISE tumors was significantly reduced in RG7112-treated mice (*P* = 0.011 and *P* = 0.016, respectively), indicating that RG7112 inhibits angiogenesis (Figure 5C and Supplementary Figure 7C).

## DISCUSSION

In this study, we found that abundant expression of *MDM2* was significantly associated with poor prognosis (PFS and OS) in ovarian clear cell carcinomas, and that the MDM2 inhibitor RG7112 has *in vitro* and *in vivo* antitumor and proapoptotic activities in clear cell carcinomas without *TP53* mutations. MDM2 is a major ubiquitin ligase that cooperates with MDM4 to degrade TP53 via the ubiquitin-proteasome system [21, 32, 33]. MDM2 is negatively regulated by specific microRNAs such as miR-1827 and miR-340 [34–36], and overexpression via multiple mechanisms is observed in various types of human malignancies, including sarcomas, gliomas, hematological malignancies, and breast cancer [34, 37]. In particular, MDM2 overexpression is more likely to occur in cancers without *TP53* mutations [37], as *MDM2* also contains TP53-binding sites in the first intron, and is thus directly inducible by wild type TP53 [34]. Accordingly, MDM2 expression was significantly lower in high-grade serous and clear cell carcinomas with mutated *TP53*. Thus, the prognostic value of *MDM2* has attracted much attention. For instance, *MDM2* overexpression was associated with poor prognosis in renal cell carcinomas [38]. In line with this result, we found that *MDM2* overexpression is an independent marker of poor prognosis in clear cell carcinomas. Importantly, *MDM2* overexpression and subsequent inactivation of *TP53* may be associated with chemoresistance [39, 40]. Taken together, the data provide a strong rationale to target MDM2 in clear cell carcinoma.

RG7112, Nutlin-3a, and RG7337 are potent and selective MDM2 antagonists, which inhibit the interaction between TP53 and MDM2, and thereby stabilizes TP53 [27, 41, 42]. We found that RG7112 has antitumor activity exclusively in clear cell tumors with wild type *TP53*. *In vitro*, the inhibitor increased the number of cells in sub-G1 phase. *In vivo*, the compound induced apoptosis by stimulating phosphorylation of TP53 at Ser-46, inducing expression of the proapoptotic TP53 target genes *PUMA* and *BAX*, and suppressing the antiapoptotic protein survivin. Although both cell-cycle arrest and apoptotic cell death can be induced by RG7112, the mechanism underlying this “switch” (from cytostatic to cytotoxic effect) has not been elucidated [27, 43]. We previously demonstrated that phosphorylation at Ser-46 is required for TP53-dependent apoptosis, and is induced by either overexpression of *TP53* or by severe DNA damage [16]. Recently, inhibition of MDM2 was reported to induce p53-induced senescence or quiescence in cells with activation of the mTOR (mammalian target of rapamycin) pathway [44, 45]. Thus, depending on TP53 status, the cytostatic or cytotoxic effects of RG7122 may vary among tumor types and even among cells in cancers with heterogeneous cell populations.

RG7112 antitumor activity was also observed in xenograft mouse models. This activity might be partly independent of apoptosis, as induction of TP21 suggests that cell cycle arrest is an underlying mechanism, as is suppression of tumor vascularization. In this study, RG7112 inhibited tumor vascularization by stabilization of TP53, which is known to prevent angiogenesis via suppression of HIF-1alpha [46]. We note that HIF-1alpha expression is significantly elevated in clear cell carcinomas compared to other types of ovarian cancers [47, 48]. Taken together, the data suggest that RG7112 may play multiple roles in inhibiting the progression of clear cell tumors.

Overexpression of *MDM4* has also been reported in various cancers, and MDM4 inhibitors were recently developed along with dual inhibitors against MDM2 and MDM4 [49, 50]. However, we found that *MDM4* is expressed only in low levels in clear cell carcinomas. Moreover, knockdown of MDM2 by siRNA clearly suppressed cell viability and induced apoptosis. Therefore, MDM2 might be a more suitable target than MDM4 in clear cell carcinomas. RG7112 is currently in phase I/II clinical trials against several cancers, but not against clear cell carcinomas. RG7112 might prove to be particularly promising against clear cell carcinoma.

This study has several limitations. For instance, the mechanisms of *MDM2* overexpression are yet to be elucidated. As MDM2 is also expressed in normal cells, the feasibility, pharmacokinetics, and pharmacodynamics of RG7112 should be carefully considered in any potential clinical application. In addition, it is unclear whether the presence or absence of TP53 mutations is a sufficient biomarker by itself to predict sensitivity to MDM2 inhibitors. Finally, further studies are needed to establish whether MDM2 abundance is associated with sensitivity to RG7112.

In conclusion, we demonstrated that *MDM2* is frequently overexpressed in clear cell carcinomas, and that *MDM2* overexpression is associated with poor prognosis. An MDM2 inhibitor, RG7112, significantly suppressed the growth of clear cell tumors with wild type TP53 both *in vitro* and *in vivo*. Furthermore, RG7112 elicited apoptotic cell death via phosphorylation of TP53 at Ser-46, and via induction of proapoptotic TP53 target genes. These findings suggest that MDM2 inhibitors such as RG7112 may be potent, targeted therapies against clear cell carcinomas.

## MATERIALS AND METHODS

### Tumor samples

Surgical samples were obtained with written informed consent from 91 patients at the University of Tokyo Hospital and Saitama Medical University International Medical Center, of whom 75 had clear cell carcinoma and 16 had high-grade serous carcinoma. The high proportion of carcinoma cells (>50%) in each sample was confirmed by a pathologist as described previously [51]. All 91 patients received primary surgery followed by 6-8 cycles of chemotherapy with paclitaxel and carboplatin. RNA was extracted and hybridized as described previously to HG-U133 Plus 2.0 Arrays (Affymetrix) containing 54,675 probes for human genes [51]. Microarray data for 41 patients (25 clear cell and 16 high-grade serous carcinomas) are available from Gene Expression Omnibus under accession number GSE65986, and 50 clear cell carcinomas were added in this study. We observed no significant biases between previous and additional microarray datasets. Patient characteristics are listed in Supplementary Table 1. Genomic DNA was extracted from 72 of 75 clear cell carcinomas, and *TP53* mutations (all exons) were determined by Sanger sequencing [52]. Total RNA from 13 normal tissues were purchased from Clontech (Mountain View, CA, USA), Ambion (Waltham, MA, USA), and Stratagene (Cedar Creek, TX, USA).

### RG7112 and cell lines

RG7112 was purchased from MedChem Express (Monmouth Junction, NJ, USA) and APEXBIO (Houston, TX, USA). OVISE, OVTOKO, and RMG-I cell lines were purchased from the Japanese Collection of Research Bioresources Cell Bank (Ibaraki, Osaka, Japan), while cell lines JHOC-7 and JHOC-9 were purchased from RIKEN Cell Bank (Ibaraki, Tsukuba, Japan). ES-2 and SKOV3 cell lines were purchased from American Type Culture Collection (ATCC) (Manassas, VA, USA). All these 7 cell lines are classified histologically as clear cell carcinoma, were cultured as described previously [48]. An immortalized cell line derived from ovarian surface epithelium was a generous gift from Dr. Hidetaka Katabuchi [53], and COS-7, and 293T cell lines were purchased from ATCC. The cell lines were authenticated by short tandem repeats.

### Proliferation assays

Cell proliferation was measured using Cell Counting Kit-8, which is based on the tetrazolium salt WST-8 (Dojindo; Minatoku, Tokyo, Japan) derived from methyl thiazolyl tetrazolium (MTT). Briefly, cells were seeded in 96-well plates at 2,000 cells/well. After 24 h, cells were incubated with 0-10 μM RG7112 for 72 h. Proliferation was quantified by monitoring the absorbance at 450 nm using a microplate reader (Bio Tek; Winooski, VT, USA). Cell proliferation was normalized relative to cell cultures treated with DMSO. Experiments were repeated at least three times.

### Immunoblotting

Cells were treated with RG7112 for the indicated time at indicated concentrations, and then lysed as described previously [54]. Lysates were probed with antibodies specific for MDM2, TP21, BAX, TP53 (Santa Cruz; Dallas, TX, USA), HIF-1alpha (Becton, Dickinson and Company; Franklin Lakes, NJ, USA), phosphorylated TP53 (Ser46), phosphorylated TP53 (Ser15), cleaved PARP, PUMA (Cell Signaling Technology; Danvers, MA, USA), survivin (Novus Biologicals; Littleton, CO, USA), and beta-actin (Sigma-Aldrich; St. Louis, MO, USA).

### Cell cycle analysis

Cells (5 × 10^5^) were seeded in 60-mm dishes and treated with RG7112 for 48 h. Floating and adherent cells were collected by trypsinization, washed twice with phosphate-buffered saline (PBS), resuspended in cold 70% ethanol, and incubated at 4°C overnight. Cells were then washed another two times with PBS, treated with 0.25 mg/mL RNase A (Sigma-Aldrich) for 30 min at 37°C, stained with 50 μg/mL propidium iodide (Sigma-Aldrich) at 4°C for 30 min in the dark, and analyzed on FACS Calibur HG (Becton, Dickinson and Company). Data were analyzed in CELL Quest pro ver. 3.1. (Becton, Dickinson and Company), and experiments were repeated three times.

### Detection of apoptosis

Cells (5 × 10^5^) were seeded in 60-mm plates for 24 h, and exposed to either DMSO or RG7112 for 72 h. Cells were then trypsinized, washed twice, doubly stained with annexin V-fluorescein isothiocynate and propidium iodide according to the manufacturer's instructions (Annexin V-FITC Apoptosis Detection Kit; BD Biosciences), and analyzed by flow cytometry. Experiments were repeated thrice.

### Gene silencing

Small interfering RNAs (siRNAs) specific for MDM2 (Stealth siRNAs HSS142909 and HSS142911) were purchased from Invitrogen (Carlsbad, CA, USA). Cells were plated in 100-mm plates at approximately 30% confluence, incubated for 24 h, and transfected for 72 h with 20 nM siRNA duplexes in Lipofectamine RNAiMAX (Invitrogen) and Opti-MEM medium (Life Technologies; Carlsbad, CA, USA). A negative control kit (Invitrogen) was used for comparison.

### Tumor xenografts in nude mice

Specific pathogen-free female nude mice (BALB/cAJc1-nu/nu) were purchased from CLEA Japan, Inc. (Meguro, Tokyo, Japan). At 5-6 weeks of age, mice were injected subcutaneously into the flank with 10^7^ RMG-I or OVISE cells in 200 μL PBS. Tumors were harvested after exponential growth, minced into 3-mm pieces, and transplanted subcutaneously into the right flank of other mice. After tumors had grown to 5 mm in diameter, mice were randomly assigned into two groups of 5-6 animals. One group received daily oral injections (100 mg/kg) of RG7112 suspended in 0.5 w/v% methyl cellulose 400 (Wako Pure Chemical Industries; Osaka, Osaka, Japan), while the other received similar doses of methyl cellulose. Tumor size was measured with a digital caliper three times weekly to estimate tumor volume according to the formula ([major axis] × [minor axis]^2^)/2. Mice were sacrificed humanely after 20 days of treatments, and tumors were preserved in 4% paraformaldehyde for immunoblotting analysis.

### Immunohistochemistry

To immunostain CD31, tumor sections were fixed at 4°C for 10 min in 4% paraformaldehyde, immersed in 1% H_2_O_2_ at room temperature for 30 min to quench endogenous peroxidases, and blocked at room temperature for 30 min with Blocking One (Nacalai tesque; Kyoto, Japan). Sections were then probed at 4°C overnight with 1:500 anti-CD31 (PECAM-1; BD Biosciences; Franklin Lakes, NJ), washed in Tris-buffered saline, and labeled at room temperature for 45 min with 1:400 biotinylated rabbit anti-rat (DAKO), and then at room temperature for 45 min with LSAB (DAKO). Finally, sections were stained with 3,3-diaminobenzidine (DAKO) and hematoxylin (Wako).

### Quantification of microvascularization

Harvested subcutaneous tumors were immunostained with anti-mouse CD31 (PECAM-1; BD Biosciences). The number of microvessels stained was counted at 400× in four random fields in each tumor.

### Statistical analysis

Survival was analyzed by Kaplan-Meier method and log rank test. Univariate and multivariate analyses were performed using the Cox proportional hazard model. Groups were compared by t-test. Data were analyzed in JMP v11 (Cary, NC, USA) and GraphPad Prism 6 (La Jolla, CA, USA), with *P* < 0.05 considered to be significant.

## SUPPLEMENTARY FIGURES AND TABLE




