# Supplementary material for: MDM2 is a potential therapeutic target and prognostic factor for ovarian clear cell carcinomas with wild type TP53
Source: Oncotarget. 2016 Sep 21;7(46):75328–38. doi: 10.18632/oncotarget.12175 (PMC5342744; doi:10.18632/oncotarget.12175)
Supplement: Supplementary file 1 [file oncotarget-07-75328-s001.pdf]

MDM2 is a potential therapeutic target and prognostic factor for ovarian clear cell carcinomas with wild type TP53

SUPPLEMENTARY FIGURES AND TABLE

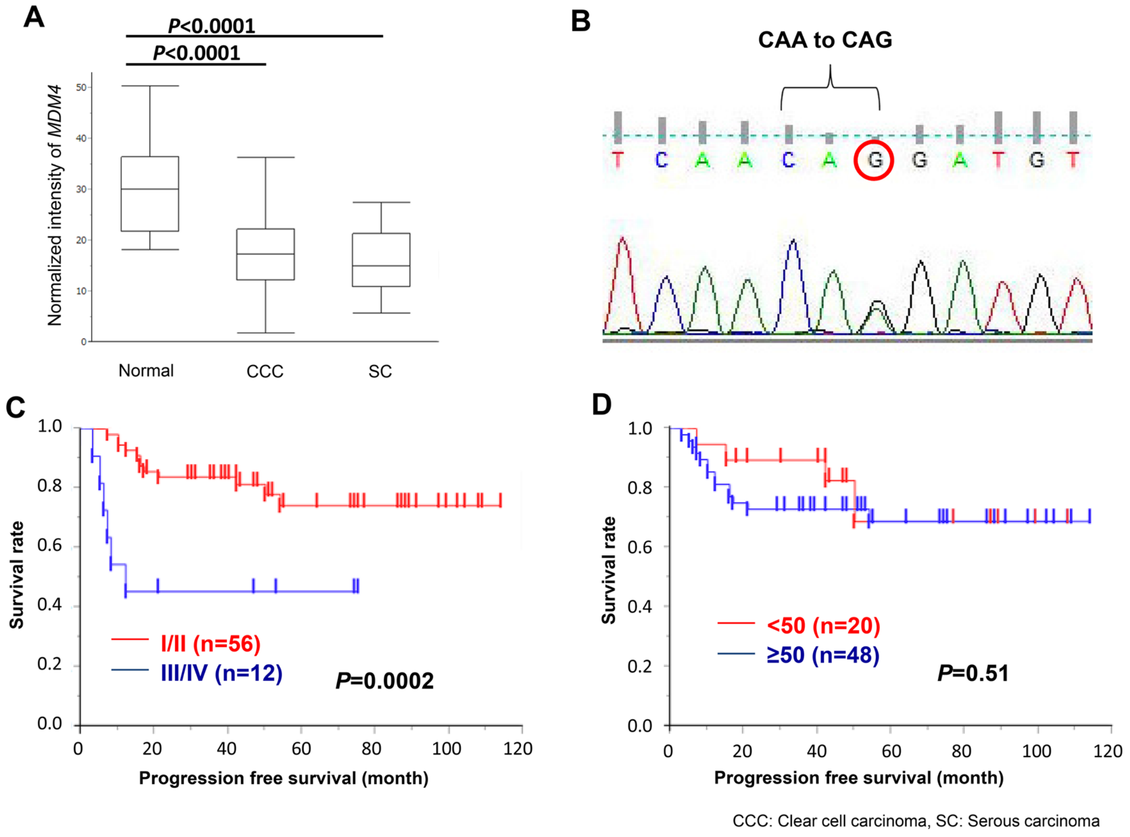

Supplementary Figure 1: Expression of MDM4 in normal tissues and ovarian cancers, identification of TP53 mutations, and progression-free survival (PFS) analysis. A. Comparison (t-test) of *MDM4* expression in normal tissues (n=13) and clear cell (n=75), and high-grade serous carcinomas (n=16). **B.** Identification of *TP53* mutation (A395G, K132R) by Sanger sequencing. **C** and **D.** Kaplan-Meier analysis of clear cell carcinomas by stage (C) and age (D)

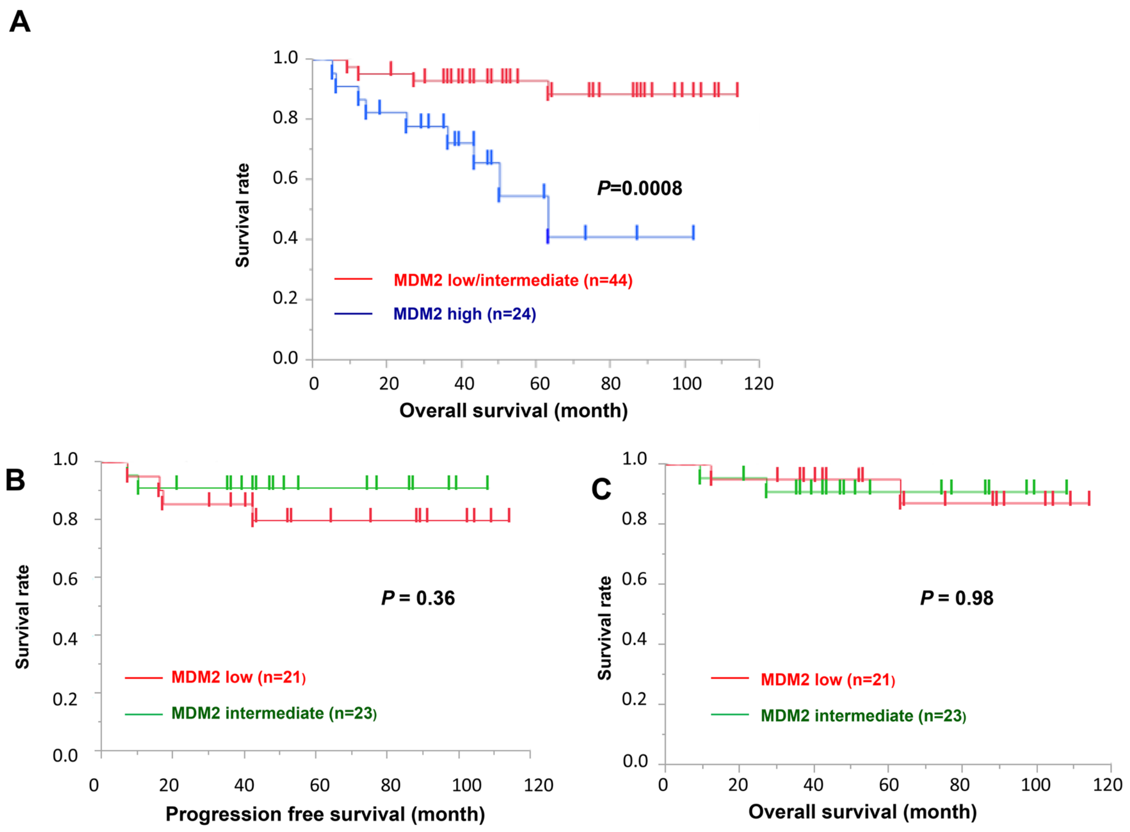

Supplementary Figure 2: Expression of MDM2 and prognosis in clear cell carcinomas. A. Comparison of overall survival (OS) between MDM2-high group and MDM2-low/intermediate group by Kaplan-Meier analysis. **B** and **C.** Comparison of PFS (B) and OS (C) among MDM2-high, -intermediate, and -low groups. PFS and OS were not significantly distinct between the MDM2-intermediate and MDM2-low groups.

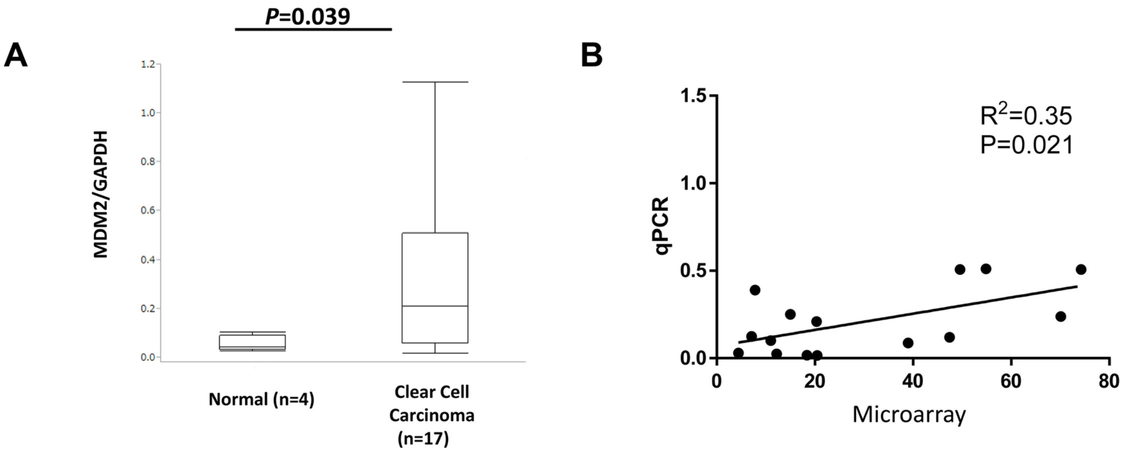

Supplementary Figure 3: Expression of MDM2 determined by real-time PCR (qPCR) in clinical samples. A. Comparison (t-test) of *MDM2* expression between 4 normal ovarian tissues and 17 clear cell carcinomas. **B.** Correlation analysis between qPCR and microarray data in the assessment of *MDM2* expression in clear cell carcinomas. Two of 17 samples were excluded as outliers. The R squared value was calculated by the Pearson correlation test, and the P value was determined by two-tailed t-test.

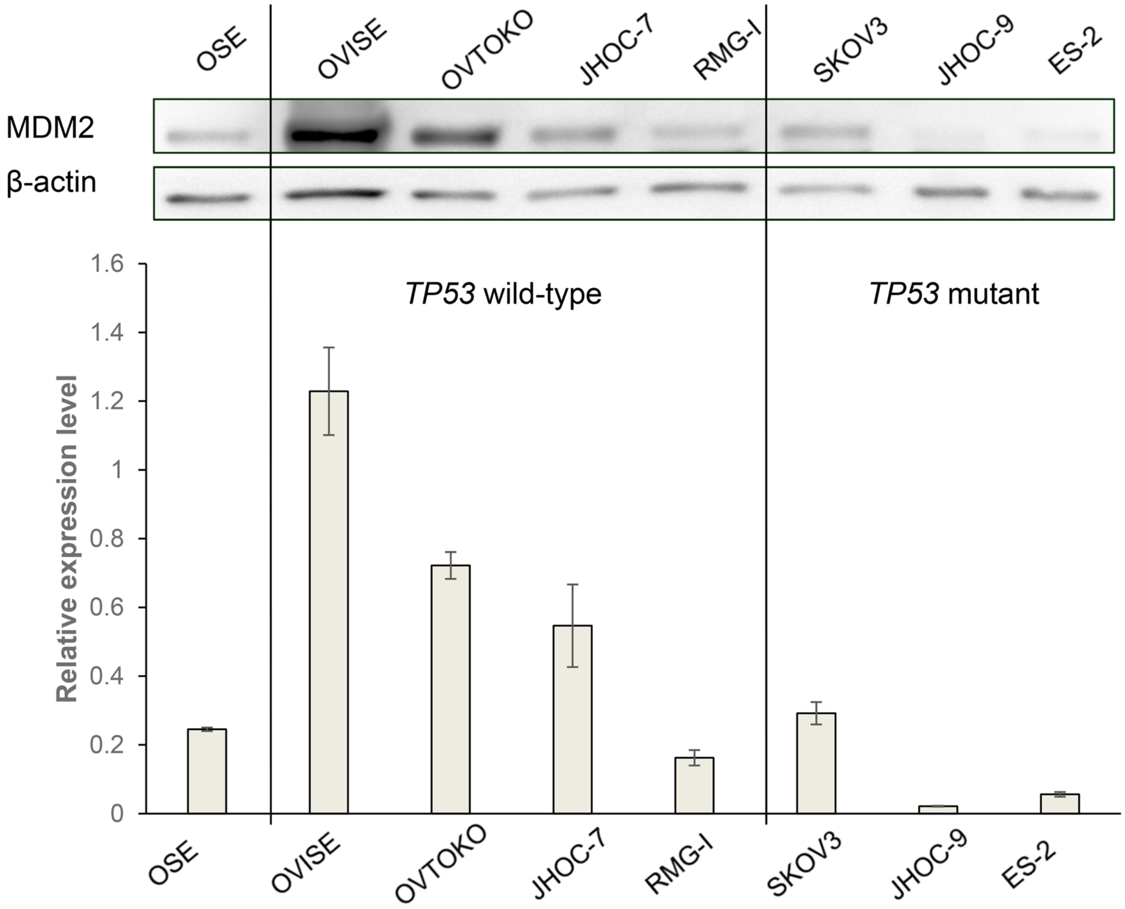

Supplementary Figure 4: Expression of MDM2 in clear cell carcinoma cell lines determined by western blotting. An immortalized cell line from ovarian surface epithelium (OSE) was used as a control. Expression of each protein relative to beta-actin was quantified in Image J. All experiments were repeated three times.

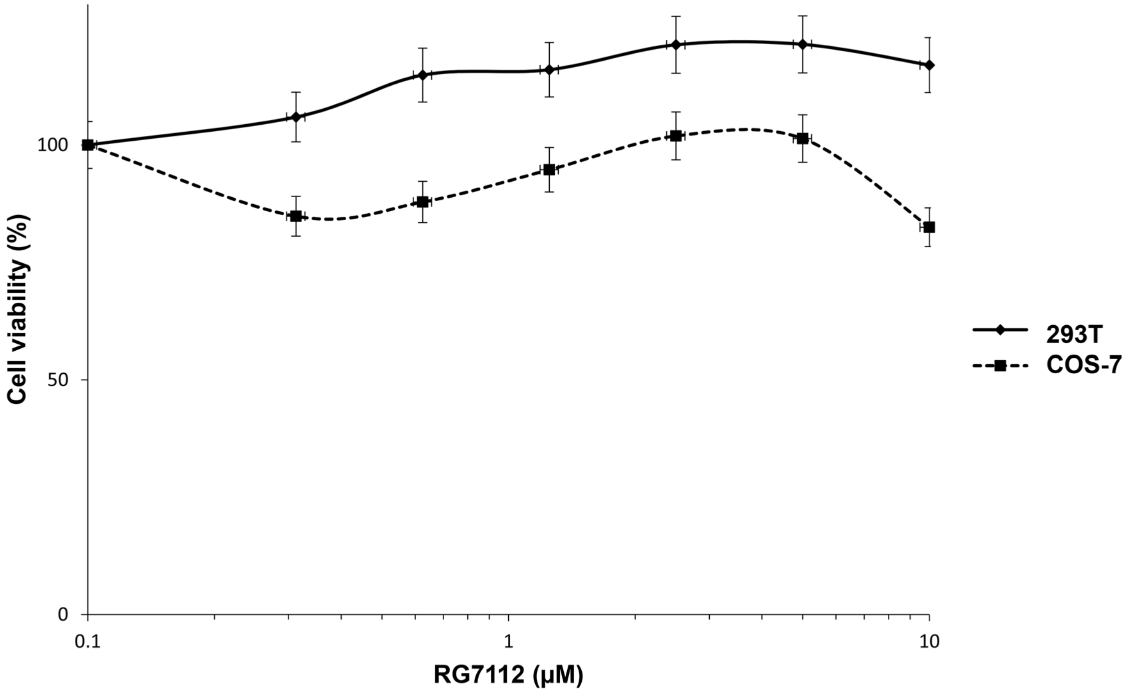

Supplementary Figure 5: Cell viability, as measured by MTT assay, in normal cells. COS-7 and 293T are derived from African Green Monkey fibroblast-like kidney cells and human embryonic kidney cells, respectively.

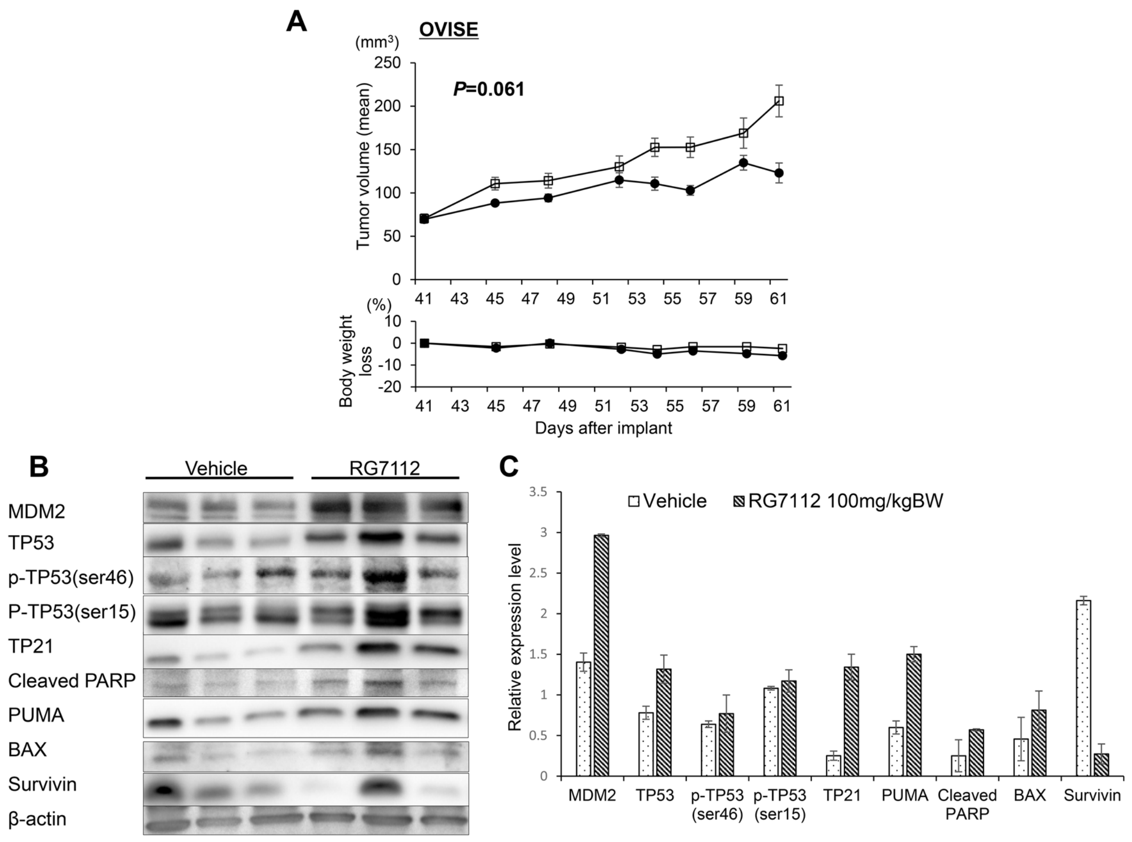

Supplementary Figure 6: *in vivo* evaluation of efficacy of RG7112 using xenografted OVISE cells. A. Tumor size and body weight in xenograft mouse models orally treated with RG7112 were measured after the start of RG7112 treatment 21 days post implantation. **B.** TP53 phosphorylation, expression of TP53 target proteins, and cleaved PARP were compared between tumors from control and RG7112-treated animals (n = 3 per group). **C.** Expression of each protein relative to beta-actin was quantified in Image J. All experiments were repeated thrice.

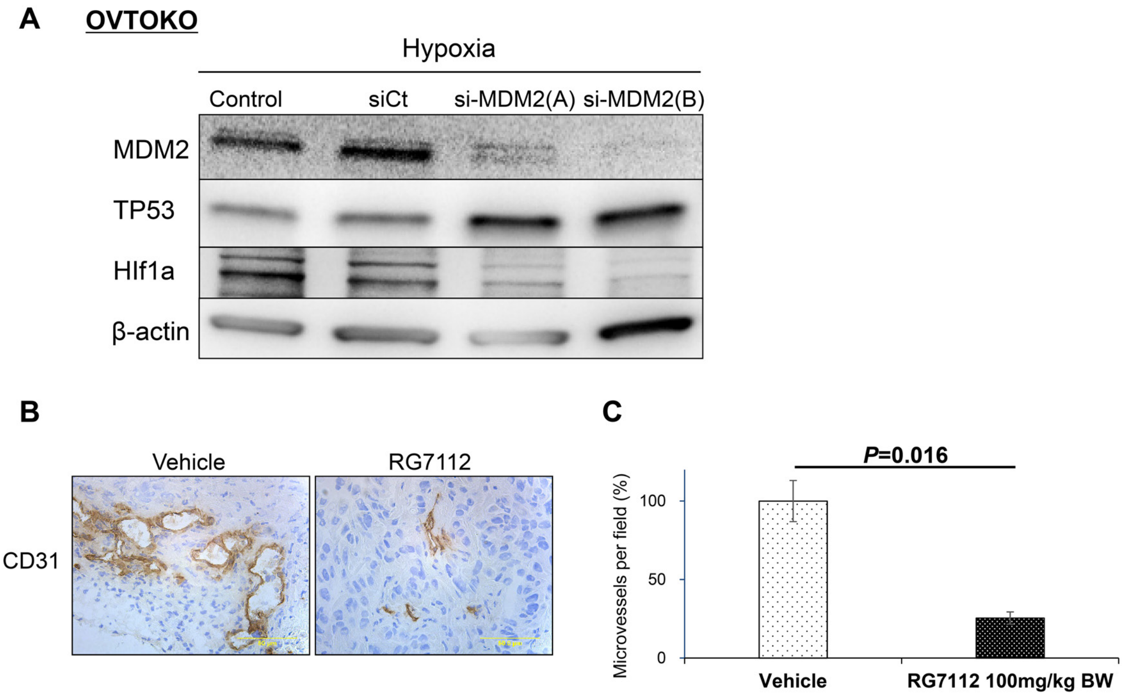

Supplementary Figure 7: Suppression of HIF-1alpha in hypoxic condition and inhibition of microvessel formation by MDM2 inhibition. A. Expression levels of MDM2, TP53, and HIF-1alpha were evaluated under hypoxic condition (1% O_2) after siRNA knockdown of MDM2 in OVTOKO cells using MDM2-A and MDM2-B. si-Ct (negative control siRNA) was used as a control. **B.** Immunohistochemical staining of CD31 to detect microvessels in OVISE tumors from control and RG7112-treated mice. **C.** Microvessels per field was calculated in OVISE tumors from control and RG7112-treated mice, and compared by t-test.

Supplementary Table 1: Patient characteristics, expression levels of MDM2/MDM4 and TP53 status in 91 ovarian carcinomas

See Supplementary File 1
